# Environmental predictors of stunting among children under-five in Somalia: cross-sectional studies from 2007 to 2010

**DOI:** 10.1186/s12889-016-3320-6

**Published:** 2016-07-28

**Authors:** Damaris K. Kinyoki, James A. Berkley, Grainne M. Moloney, Elijah O. Odundo, Ngianga-Bakwin Kandala, Abdisalan M. Noor

**Affiliations:** 1 INFORM Project, Spatial Health Metrics Group, Kenya Medical Research Institute/Wellcome Trust Research Programme, Nairobi, Kenya; 2 Kenya Medical Research Institute/Wellcome Trust Research Programme, Centre for Geographic Medicine Research (coast), Kilifi, Kenya; 3 Centre for Tropical Medicine and Global Health, Nuffield Department of Clinical Medicine, University of Oxford, CCVTM, Oxford, OX3 7LJ UK; 4 Nutrition Section, United Nations Children’s Fund (UNICEF), Kenya Country Office, UN Complex Gigiri, Nairobi, Kenya; 5 Food Security and Nutrition Analysis Unit (FSNAU) - Somalia, Food and Agriculture Organization of the United Nations, Ngecha Road Campus, Nairobi, Kenya; 6 Warwick Medical School, Health Sciences Research Institute, University of Warwick, Warwick Evidence, Gibbet Hill, CV4 7AL Coventry, UK; 7 Department of Mathematics and Information sciences, Faculty of Engineering and Environment, Northumbria University, Newcastle upon Tyne, NE1 8ST UK; 8 Department of Population Health, Luxembourg Institute of Health (LIH), 1A-B, rue Thomas Edison, L-1445 Strassen, Luxembourg

**Keywords:** Malnutrition, Stunting, Somalia, Forecasting

## Abstract

**Background:**

Stunting among children under five years old is associated with long-term effects on cognitive development, school achievement, economic productivity in adulthood and maternal reproductive outcomes. Accurate estimation of stunting and tools to forecast risk are key to planning interventions. We estimated the prevalence and distribution of stunting among children under five years in Somalia from 2007 to 2010 and explored the role of environmental covariates in its forecasting.

**Methods:**

Data from household nutritional surveys in Somalia from 2007 to 2010 with a total of 1,066 clusters covering 73,778 children were included. We developed a Bayesian hierarchical space-time model to forecast stunting by using the relationship between observed stunting and environmental covariates in the preceding years. We then applied the model coefficients to environmental covariates in subsequent years. To determine the accuracy of the forecasting, we compared this model with a model that used data from all the years with the corresponding environmental covariates.

**Results:**

Rainfall (OR = 0.994, 95 % Credible interval (CrI): 0.993, 0.995) and vegetation cover (OR = 0.719, 95 % CrI: 0.603, 0.858) were significant in forecasting stunting. The difference in estimates of stunting using the two approaches was less than 3 % in all the regions for all forecast years.

**Conclusion:**

Stunting in Somalia is spatially and temporally heterogeneous. Rainfall and vegetation are major drivers of these variations. The use of environmental covariates for forecasting of stunting is a potentially useful and affordable tool for planning interventions to reduce the high burden of malnutrition in Somalia.

**Electronic supplementary material:**

The online version of this article (doi:10.1186/s12889-016-3320-6) contains supplementary material, which is available to authorized users.

## Background

Stunting, defined as linear growth failure is a result of chronic inadequate nutrition, infections or environmental-triggered intestinal inflammation [1–3]. The World Health Organization (WHO) classifies a child as stunted if his or her length- or height-for-age z-score is below −2 [4, 5]. Stunting among children under the age of five years has long-term effects on cognitive development, school achievement, economic productivity in adulthood and maternal reproductive outcomes [6]. It is also recognized to have a major impact on childhood mortality, and is therefore a key concern for policy and programs addressing child survival [7].

Approximately, 39 % of children under the age of five years in the developing world are estimated to be stunted, the highest rates being in Asia and sub-Saharan Africa [8]. In particular, populations in countries with conflict suffer disruption in livelihoods, assets, nutrition and health. Consequently, conflict has enormous negative impact on food, economy, health and the environment [9]. In 2005–07 the proportion of malnourished people living in countries with a protracted crisis ranged from 14 % in Côte d’Ivoire to 69 % in the Democratic Republic of the Congo [10]. In Somalia, the national mean prevalence of stunting was estimated to be greater than 20 % [11], indicating a critical situation by WHO standards [12, 13].

A number of risk factors including infection, seasonal food insecurity and environmental conditions have been previously shown to be associated with stunting [14–17]. Rural households have been reported to be predominantly affected because they heavily depend on traditional rain-fed agriculture and pasture for livestock. Rainfall and vegetation therefore play a major role in determining the economic and nutrition status of the rural communities [18, 19]. These environmental conditions are influenced largely by intra-seasonal and inter-annual climatic variability resulting in extreme events such as drought and floods that reduce agricultural outputs and pasture leading to severe food shortages. Therefore monitoring environmental conditions may help in forecasting the prevalence of malnutrition especially in sub-Saharan countries where livelihoods are closely intertwined with climate variability [20]. This will help in identifying hotspots of high prevalence using the patterns of environmental and climatic determinants to guide in predicting areas where timely interventions are likely be targeted.

Previous efforts in predicting malnutrition have focused on ecological and areal data analysis [21, 22]. These methods give mean estimates at regional or district level but do not capture the variability of prevalence that occurs within regions. In this study, we used Bayesian space-time geostatistical models to determine how well environmental covariates can be used to forecast the prevalence of stunting among children under the age of five years from 2008 to 2010 in Somalia. Continuous surfaces of environmental covariates together with observed rates of stunting from the sampled locations were used to predict the rates of stunting to unsampled locations and forecast prevalence on a continuous scale at 1 × 1 km spatial resolution. This method accounts for heterogeneity in prevalence influenced by factors such as precipitation and vegetation as well as proximal factors to give accurate estimates at a high spatial resolution [23].

## Methods

### Somalia country context

Somalia has a population of approximately 12 million people with an estimated 1.7 million children under the age of five years. Child mortality rate is estimated to be 180 per 1000 live births, which translates to approximately 71,000 deaths annually in children under the age of five years [24].

Pastoralism and agro-pastoralism are the two dominant livelihood systems in Somalia. Pastoralists are mainly rural and predominantly in the arid areas of northern and central Somalia, as well as along Ethiopian and Kenyan borders. Agro-pastoralists communities practice mixed agriculture in the marginal lands [25], and are found in the inter-riverine regions of Bay, Bakool, western Hiraan and eastern Gedo in Southern Somalia, and in small areas of the Northern regions [25]. A small proportion of the riverine population along the Juba and Shabelle rivers depends on settled agriculture. Fishing represents only a very small livelihood activity, despite Somalia having one of the longest coastlines in Africa [25]. The Somali population relies on long rains, the *Gu* in April to June and the shorter rains, *Deyr* in October to December for agricultural production, pasture regeneration and replenishment of rivers, dams and ground water supply. However, recurrent droughts and flooding in the country has affected the livelihoods, food security and nutritional status [25].

Due to inadequate governance structures in parts of Somalia because of the long civil war and consequent insecurity, nutrition planning and response is mainly undertaken by international organizations in a ‘Nutrition Cluster’ formed in 2006 [25]. So far, nutrition responses have primarily focused on intervening against the alarming rates of acute malnutrition throughout the country. Several feeding programmes for severe acute malnutrition are implemented across Somalia by UN agencies, international and local non-governmental organizations (NGOs). However the coverage and quality of interventions is limited by the overall weakness of the public health system and poor accessibility to several parts of the country [26].

### Survey data

The data used for this study were obtained from the Food Security and Nutrition Analysis Unit (FSNAU) of the Food and Agriculture Organization (FAO) and are from surveys undertaken between 2007 and 2010. During this period, FSNAU, in partnership with United Nations Children’s Fund (UNICEF), conducted bi-annual seasonal nutrition assessment surveys using standard methods, indicators and tools [26–28]. Sample size was calculated for children aged 6–59 months using Epi Info/ENA 2008 software (Center for Disease Control (CDC) in USA), the sampling unit being the household [27, 28]. This methodology used estimated average household size and the proportion of children aged 6–59 months in the population obtained from previous surveys or national statistics. Detailed description of the survey methods are described elsewhere [27].

Spatial coordinates for each survey cluster were acquired through an online search and verified using Google Earth (Google, Seattle, USA) to confirm if the coordinates matched evidence of human settlement. Those settlements for which no reliable source of coordinates could be obtained were excluded from the analysis. These data were then aggregated at the cluster level with the corresponding geographical covariates and year of survey. Each record represented a cluster and consisted of total children examined, stunted children, a list of geographical covariates and the year and season of the survey. Table 1 provides a detailed summary of the data used in the study.

**Table 1:** A summary of the survey data used in this study by zone and region in Somalia. Children with height-for-age z-scores of < −2 were considered stunted according tothe WHO growth standards [4, 5]

Zone	Region	Number of clusters	Number of children examined	Number of children stunted	Percent stunted
North East (Puntland)	Bari	9	756	201	26.59
	Mudug	61	6188	804	12.99
	Nugaal	24	1673	383	22.89
North West (Somaliland)	Awdal	26	862	7	0.81
	Sanaag	14	412	3	0.73
	Sool	3	142	18	12.68
	Togdheer	12	673	362	53.79
	Woqooyi Galbeed	23	2465	1378	55.90
South Central	Bakool	75	3534	1150	32.54
	Banadir	1	51	0	0.00
	Bay	98	5568	2133	38.31
	Galgaduud	77	5831	1908	32.72
	Gedo	111	6985	1999	28.62
	Hiraan	142	10743	2260	21.04
	Juba Dhexe	77	5253	2734	52.05
	Juba Hoose	71	5560	1553	27.93
	Shabelle Dhexe	101	7650	2414	31.56
	Shabelle Hoose	141	9432	3432	36.39
Total		1066	73778	22739	30.82

### Statistical analysis

The first step of the analysis involved examining the effects and the patterns of five environmental covariates: precipitation, enhanced vegetation index (EVI), temperature, distance to main water sources and urbanization. These covariates were selected based on their association with vector-borne diseases and food security [29, 30]. The effects of the covariates were determined using a Bayesian Information Criterion (BIC) through generalized linear approach [31]. Bayesian hierarchical space-time models were implemented using integrated nested Laplace approximations (INLA) in R-INLA library [32] to predict on continuous maps the prevalence of stunting at 1 × 1 km spatial resolution using the data and selected covariates [33]. The prediction of the prevalence of stunting was done using two models. In the first model (model 1), the prevalence of stunting was forecast for a future year by using the environmental covariates for this year and the coefficients derived from a regression analysis of the relationship between stunting and the environmental covariates of the preceding years using a geostatistical model. For example, the forecasting for 2008 was made using the regression coefficients of 2007 survey data and environmental covariates applied to the 2008 environmental covariates, while 2010 forecast was based on the regression coefficients of the space-time geostatistical model that used the survey data and environmental covariates from 2007 to 2009 applied to the environmental covariates for 2010. In the second model (model2), the whole dataset and the environmental covariates from all the years were used to fit the model parameters and predict the prevalence of stunting from 2007 to 2010 using the space-time geostatistical model. This model was considered to be the gold-standard for our analysis as it used the whole data in simultaneous modelling of prevalence and the prediction of uncertainty and accounted fully for the complete spatial and temporal dependence to predict to each year from 2007 to 2010 [34]. These model results were used to validate the predictive power of model 1. The procedure was repeated for data among children aged 6–30 months to observe the variability of the rates of stunting in this age group using the two models.

The estimation stages of the two geostatistical models were implemented using Stochastic Partial Differential Equations (SPDE), which are formulated as a link between Gaussian random fields (GRFs) and the Gaussian Markov Random Fields (GMRFs) [32]. The spatiotemporal covariance function and the dense covariance matrix of the Gaussian field are replaced by a neighborhood structure and a sparse precision matrix respectively that together define a GMRF [35]. The posterior mean for each specified year of prediction was mapped at 1 × 1 km spatial resolution, which was then further classified into WHO categories of stunting among children less than 5 years of age [36]. The proportions of stunted children were computed for each region and year in Somalia. This was obtained by multiplying the posterior mean proportion of stunted children at each 1 × 1 km pixel with the corresponding population estimate [37] of children under the age of five years at the same location and year to compute the number of children stunted at each pixel. These numbers were summed for each region and then divided by the corresponding total population of under-fives for the region [37].

To assess the performance of the forecasting model, we compared the forecasted proportion of stunted children in the survey locations and the actual prevalence at these locations by randomly generating 10 % validation dataset using a sampling algorithm which declusters over space and time. Four performance indices were computed to evaluate the predictive performance and model fit for the two models: root-mean-square error (RMSE), mean prediction error (MPE), mean absolute prediction error (MAPE) and the correlation coefficient between the predicted and the observed values. We examined the correlation between the forecast and observed prevalence using correlation plots. Further, the marginal excursion probabilities for each prevalence class based on the estimated posterior distribution for stunting were simultaneously calculated using numerical integration (NI) method as implemented by Bolin and Lindgren 2012 [38]. Areas where the stochastic process exceeded prevalence of stunting of 40 % (extreme stunting according to the WHO) were then significantly determined using the positive excursion function in NI method and the parametric family of excursion sets [38]. The correlation between the full data and forecast models values from the two models were visualized using column plots at regional level for 2008 to 2010. Detailed methods on covariate selection, Bayesian hierarchical space-time modeling, validation procedures can be found in the Additional file 1.

## Results

A total of 1,066 unique survey locations sampled during the period of 2007 to 2010 were included in the analysis (Additional file 1: Figure SI 1a & 1b). About 36 % of the data was collected in 2007, 28 % in 2008, 22 % in 2009 and 14 % in 2010. The location of 1,765 children from 34 clusters could not be accurately determined and were therefore excluded from the analysis. Out of the 73,778 children included in the analysis, 22,739 (31 %) were stunted [Table 1]. Precipitation (OR = 0.994, 95 % credible interval (CrI): 0.993, 0.995) and enhanced vegetation index (EVI) (OR = 0.719, 95 % CrI: 0.603, 0.858) were the only environmental covariates that were significantly associated with stunting and were used in the space-time geo-statistical models.

**Fig. 1:**
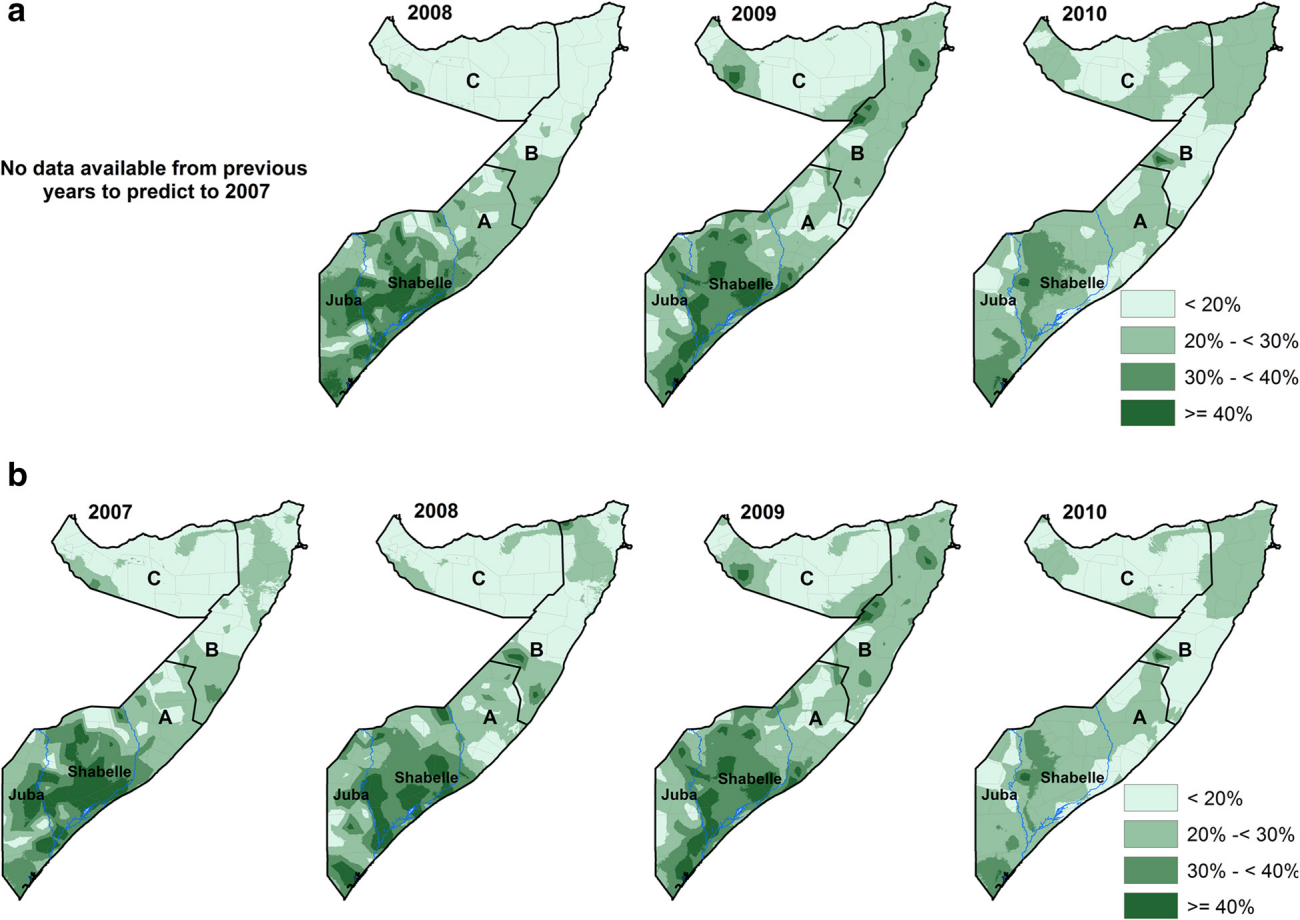
**a** Model 1 (forecast model): The predicted 1 × 1 km posterior mean stunting maps classified using WHO categories for 2008 and 2010 for children aged 6–59 months. Less than 20 % represents low prevalence; 20 % – < 30 %, medium prevalence; 30 % – < 40 %; high class and > =40 % represents the very high prevalence. **b** Model 2 (full data model): The predicted 1 × 1 km posterior binned stunting mean maps for 2007 and 2010 for children aged 6–59 months. Less than 20 % represents low prevalence; 20 % – < 30 %, medium prevalence; 30 % – < 40 %; high class and > =40 % represents the very high prevalence according to the WHO prevalence classification. A = South Central zone, B = North East (Puntland) zone, C = North West (Somaliland) zone. The *blue lines* represent the two rivers in Somalia (Juba and Shabelle)

The posterior distributions of stunting for the forecast years from the two models were displayed using WHO categories on a 1 × 1 km spatial resolution grid are shown in Fig. 1a and b. The national prevalence of stunting was estimated at 30 % in 2008, 31 % in 2009 and 28 % in 2010. The prevalence is highest in South Central zone followed by North East (Puntland) and lowest in North West (Somaliland). Additional file 1: Figure SI 2 in the supplementary file shows the continuous maps at 1 × 1 km spatial resolution of standard deviations of the posterior mean stunting for the years 2007 to 2010.

**Fig. 2:**
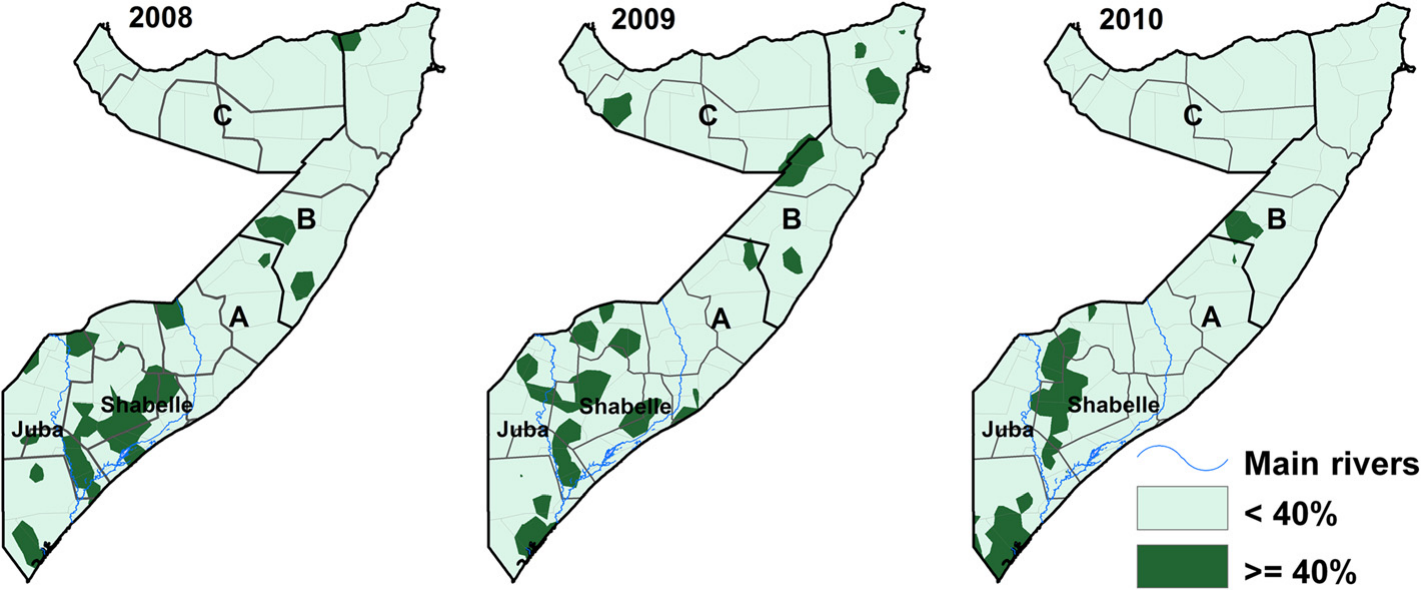
Estimated areas with exceedance probabilities of the 40 % prevalence of stunting for children aged 6–59 months using the excursion contour functions of model 1 [38]. The maps were developed from extracting the > =40 % class produced from the surfaces of the simultaneous marginal excursion probabilities. The *dark green* color shows areas that are equal to or significantly greater than 40 % prevalence of stunting. These areas are of the ‘very high’ prevalence category according to the WHO classification [4, 5]. A = South Central zone, B = North East (Puntland) zone, C = North West (Somaliland) zone. The *blue lines* represent the two rivers in Somalia (Juba and Shabelle)

Maps of marginal excursion probabilities based on the estimated posterior distribution threshold exceeding 40 % prevalence of stunting are shown in Fig. 2. The regions that consistently exceeded 40 % prevalence were Bay, Gedo, Bakool, Mudug, Lower and Upper Juba. All these regions are in the South Central zone.

**Fig. 3:**
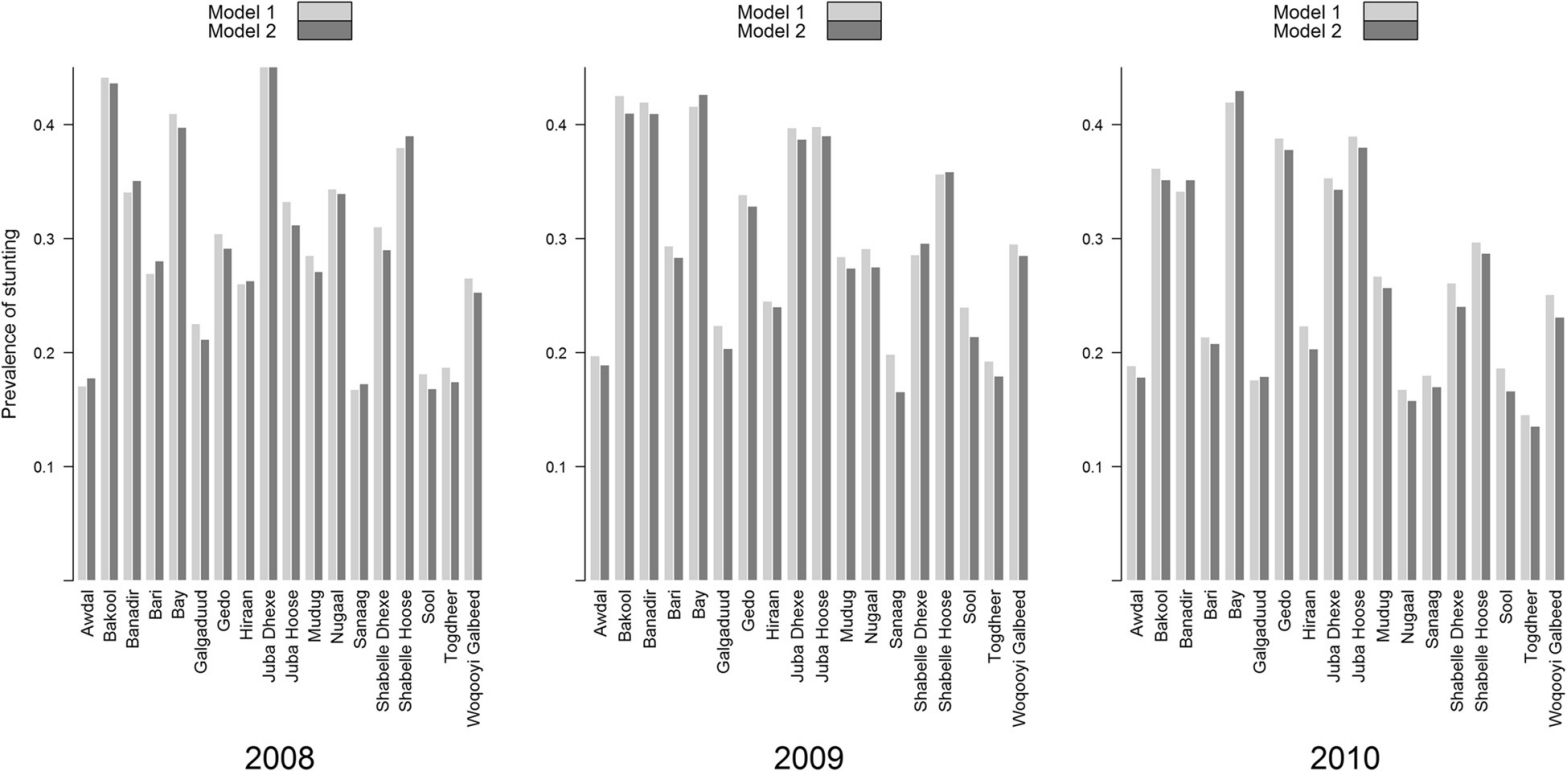
Percent population of children aged 6–59 months who were stunted in the years 2007 to 2010 by region in Somalia estimated using models 1 (forecast) and 2 (full data)

Figure 3 shows the estimated proportions of children under five years of age who were stunted by region and year of survey from models 1 (forecast) and 2 (full data). The maximum difference in the estimated prevalence of stunting between the two models was less than 3 % in all the regions across all forecast years. The highest inconsistency in prevalence of stunting between the forecast and full data models was observed in 2008, followed by 2009 and was lowest in 2010. In South Central zone, the prevalence of stunting in children was consistently high in Bay (41 %), Middle (40 %) and Lower Juba (37 %) and Lower Shabelle (39 %) for the three years of study. In the North East and North West zones, prevalence of stunting was high in Bari (26 %) and Woqooyi Galbeed (26 %) regions respectively. Detailed correlation plots of observed and predicted values at cluster level by year can be found in Additional file 1: Figure SI 3a, 3b and 3c. In addition, maps of posterior means among children aged 6–30 months from the two models can be found in Additional file 1: Figure SI 4a and 4b.

The validation statistics for the forecast model (**model 1**) in 2008, 2009 and 2010 respectively were: RMSE = 0.146, 0.127, 0.104; MPE = 0.003, 0.006, 0.019; MAPE = 0.109, 0.091, 0.071 and the correlation coefficient between the observed and predicted were 0.619, 0.658, and 0.519 for 2008, 2009 and 2010 respectively. The validation statistics for the full space time model (**model 2**) were: RMSE = 0.136, MPE =0.002, MAPE = 0.105 and linear correlation coefficient of 0.668. Maps of the data distribution per year of study, posterior standard deviations from the mean and the correlation plot of the posterior predicted mean and observed values of the dataset can be found in the Additional file 1.

## Discussion

The aim of this study was to forecast the levels and patterns of childhood stunting in Somalia at a high spatial resolution using available survey data and standard environmental covariates. To achieve this, we modeled the changing patterns of prevalence of stunting from 2007 to 2010 using the largest available dataset of malnutrition among children under the age of five years in Somalia. The two main environmental covariates used in this study were rainfall and vegetation cover. The first model forecast the prevalence of stunting by using the regression results of observed stunting and the environmental covariates at the survey locations for a given year and then applying these regression coefficients to the environmental covariates in a subsequent year using a geostatistical model. The second model used the whole dataset and the environmental covariates from all the years to fit the model parameters and predict the prevalence of stunting in each year. The difference in the estimated prevalence of stunting in the two models was less than 3 % across all the regions and survey years in Somalia. Overall, the distribution of stunting in Somalia suggests substantial spatial heterogeneity with prevalence consistently higher in the regions of the South Central zones compared to those in the North.

Our study has shown that data on environmental changes related to the variability of rainfall and vegetation cover provide unique opportunities to predict future rates of stunting and can help identify populations that are likely to be most affected to guide interventions. Previous literature shows that spatial patterns of food insecurity in sub-Saharan Africa are correlated with the rainfall anomalies and vegetation cover [39]. Droughts that have been experienced in the Horn of Africa have directly resulted in food crises in 1984–1985, 2000–2001 and 2002–2003 and caused widespread famine in 25 African countries [39]. For example, the drought in Ethiopia in 2003 affected approximately 13 million people [40]. In 2011, some parts of southern Somalia were affected by famine in which approximately 4 million people, nearly half the country’s population, faced a humanitarian crisis [41]. In West African countries, the intensity of poverty was shown to be inversely associated with the vegetation cover [42].

A potential reason for high levels of stunting in Somalia is the failure of seasonal rains for a long time which have caused a severe water crisis in most parts of the country with the exception of the North West zone [43]. Agro-pastoral and riverine livelihoods found in South Central zone mainly depend on rainfall and have repeatedly been subjected to dry conditions since 2007 which resulted in a significant crop harvest failure [43]. This led to dramatic increases in the price of water and local cereals, which were the main drivers of a deteriorating food security situation in Somalia [43]. The pastoralist communities, predominantly found in the northern region, have also been severely affected by the water crisis caused largely by the failed rains. As a coping mechanism, the pastoral communities migrate seasonally in search for water and pasture thereby reducing the effects of droughts, however, the most threatened are those with cattle and sheep because these herds have limited ability to migrate for long distances [12, 44, 45].

The sustained conflict in Somalia has led to lack of reliable ‘safety nets’ and appropriate financial support mechanism. This has necessitated the distribution of humanitarian aid, including food supplies, to strengthen households coping mechanisms [18]. It is unclear, however, if these interventions have achieved the right impact and whether mitigation of the effects of immediate shocks have led to strengthening household capacity for self-sustenance after the shock. Persistent conflict has also hindered access to credible information on the burden of malnutrition that can be used to establish public health priorities and develop timely interventions in the country. Our study shows that with improved understanding of climate variability, the implications of weather patterns for the food security and vulnerability of rural communities can be more predictable and be monitored effectively.

Our analysis has some limitations. First, the use of the WHO reference population for measuring stunting among Somali children may be problematic. Often, Somali babies have lower weight at birth, which may be thinness more than shortness [46]. In addition, the average Somali child is thinner and taller, by 12–24 months, with half the stunting prevalence (defined by height-for age) when compared to other children in the neighboring East African countries [46]. It is also noted that children’s growth patterns in pastoralist communities differ considerably from those of children in populations with agricultural livelihoods where pastoral communities demonstrate decreasing weight-for-height with increasing age which leads to tall but light in body-weight in adulthood [47]. The WHO standards may therefore underestimate stunting in most of Somalia. There is also uncertainty in determining child age among rural populations where there are limited access to antenatal services and therefore low possession of health or immunization cards [48, 49]. In our study, the age of the child was provided mainly by their mothers often without any means of verifying the data. The sustained conflict would have exacerbated malnutrition in Somalia and continues to be the primary reason for population displacement in the South Central zone. However, in this study, reliable conflict data was not available at the time of analysis and was not accounted in the modeling process. Although the performance of the forecast and full data models varied slightly, our models identified extreme values as outliers, which were therefore normalized. However, the models also suggest the presence of hotspots of stunting that vary between years and these may be partly linked to variations in the sampling of survey locations during each cross-sectional survey.

## Conclusion

The rate of stunting in Somalia is spatially and temporally heterogeneous and rainfall and vegetation are major drivers of these variations. The use of environmental covariates as alternative to surveys or complementary to sparse data can help forecast stunting and inform programme preparedness. Nutrition responses in Somalia are primarily focused on responding to the alarming rates of acute malnutrition. It has been estimated that if a package of nutrition-specific intervention that includes management of acute malnutrition and supplementation of multiple micronutrients is scaled up to 90 % coverage, stunting would be reduced by 20 % and this would reduce under-five mortality by 15 % [17, 50]. Our findings, especially using the maps that use the environmental data, may help programmes to target these interventions at high spatial resolution to improve efficiency.

## Abbreviations

CDC, center for disease control and prevention; EVI, enhanced vegetation index; FAO, food and agriculture organization; FSNAU, food security and nutrition analysis unit; GMRFs, gaussian markov random fields; GRFs, Gaussian random fields; INLA, integrated nested laplace approximations; MAPE, mean absolute prediction error; MPE, mean prediction error; NGOs, non-governmental organizations; NI, numerical integration; RMSE, root-mean-square error; SPDE, stochastic partial differential equations; UNICEF, United Nations Children’s Fund; WHO, World Health Organization

## Additional file

Additional file 1: Environmental predictors of stunting in Somalia – a geostatistical approach. (DOCX 1622 kb)
