# Supplementary material for: Environmental predictors of stunting among children under-five in Somalia: cross-sectional studies from 2007 to 2010
Source: BMC Public Health. 2016 Jul 28;16:654. doi: 10.1186/s12889-016-3320-6 (PMC4963948; doi:10.1186/s12889-016-3320-6)
Supplement: Additional file 1: — Environmental predictors of stunting in Somalia – a geostatistical approach. (DOCX 1622 kb) [file 12889_2016_3320_MOESM1_ESM.docx]

***Environmental predictors of stunting in Somalia – a geostatistical approach***

**SI 1: Covariate processing and selection**

A set of five geographical covariates were examined; Precipitation, Enhanced Vegetation Index (EVI), Temperature, Distance to water bodies and Urbanization. Precipitation and temperature rasters were derived from the monthly average rasters obtained from WorldClim website and were summarized to get the mean rainfall and mean temperature surfaces by year[1]. The EVI surface was derived from the MODerate-resolution Imaging Spectroradiometer (MODIS) sensor imagery[2] while the urbanization surface was obtained from Global Rural Urban Mapping Project (GRUMP)[3].

We began the process of covariate selection with extracting the values of the selected covariates from the geographical surfaces to survey locations in the dataset using ArcGIS 10 Spatial Analyst (ESRI Inc. NY, USA) tool corresponding to the year of survey. Then best generalized linear approach was used to generate minimum adequate set of covariates that have a significant effect on stunting to be used in geostatistical model. The function “bestglm” was used as implemented in R-Project version 3.0.1 package. This function selects the best subset of the input covariates for the GLM family[4]. We used the Bayes Information Criterion (BIC) to select the significant covariates for the study because it has been shown that BIC often selects more parsimonious models than the AIC[4]. A uniform prior of the model of fixed size implemented in $BIC_{\gamma}$ was used (Equation 1).

$BIC_{\gamma}=D+k\log(\mathrm{n})+2\gamma\log\left(\begin{matrix} p \\ k \end{matrix}\right)$ (1)

This is where $\gamma$ is an adjustable parameter, p is the number of possible input covariates not counting the bias or intercept term and $k$ is the number of the parameters in the model[4].

**SI 2: Space-time Bayesian Geo-statistical model**

We implemented a Bayesian hierarchical space-time model through SPDE approach using R-INLA library to produce continuous maps of the risk of stunting at 1 x 1 km spatial resolution and predicting to each year of study from 2008 to 2010 for model 1 and 2007 to 2010 for model 2[5]. This SPDE is formulated as a link between Gaussian random fields (GRFs) and the Gaussian Markov Random Fields (GMRFs)[6]. The spatio-temporal covariance function and the dense covariance matrix of the Gaussian field are replaced by a neighborhood structure and a sparse precision matrix respectively that together define a GMRF[7]. This finite-dimensional GMRF that substitutes infinite-dimensional GRF can be expressed as shown in Equation 2.

$x(u)=\sum_{i=1}^{n} \psi_{i}(u)w_{i}$ (2)

Here the $\left\{w_{i}\right\}$ represents the Gaussian distributed weights and $\Psi_{i}$ are piece-wise linear basis functions defined on a triangulation of the domain with $n$ nodes defined as mesh. The solution of a Gaussian random field SPDE with Matern covariance function is represented as in Equation 3.

$(\mathrm{k}^{2}-\Delta)^{\alpha/2}x(\mathrm{u})=W(\mathrm{u}),$ $u\in R^{d},$ $\alpha=v+d/2,$ $k>0,$ $v>0,$ (3)

The innovation process $W$is the spatial Gaussian white noise and $\Delta$ is the Laplacian. Finite element method (FEM), a numerical technique for solving partial differential equation, has been successfully used in solving the SPDE of this type[6]. This SPDE-formulation is motivated by computational benefits and also introduces a new class of spatial models[8]. In this SPDE approach, a non-stationary model was used and achieved by modifying the SPDE to obtain the GRFs with defined dependence structure and is expressed as

$(k(\mathrm{u})^{2}-\Delta)(\tau(\mathrm{u})\mathrm{x}(\mathrm{u}))=\mathrm{w}(\mathrm{u}),$ $u\in\Omega,$ (4)

In the current version of the SPDE package as implemented in[6], a non-stationary model defined via spatial varying $k(\mathrm{u})$ and $\tau(\mathrm{u})$ is available for the case$\alpha=2$ . The $\mathrm{logk}(\mathrm{u})$ and $\log\tau(\mathrm{u})$ are defined as linear combinations of basis functions,

$\log(\tau(\mathrm{u}))=\mathrm{b}_{0}^{\tau}(\mathrm{u})+\sum_{k=1}^{p} b_{k}^{\tau}(\mathrm{u})\theta_{k},$ (5)

$$\log(\mathrm{k}(\mathrm{u}))=\mathrm{b}_{0}^{k}(\mathrm{u})+\sum_{k=1}^{p} b_{k}^{k}(\mathrm{u})\theta_{k},$$

The precision matrix with parameter fields in the diagonal matrices is evaluated in a mesh as

$T=diag(\tau(\mathrm{u}_{i})),$ $K=diag(\mathrm{k}(\mathrm{u}_{i})),$ (6)

$$Q=T(\mathrm{K}^{2}\mathrm{CK}^{2}+\mathrm{K}^{2}\mathrm{G}_{1}+\mathrm{G}_{1}^{T}\mathrm{K}^{2}+\mathrm{G}_{2})\mathrm{T}$$

The space-time SPDE model used in this study is represented as show in equation 7. This is by constructing Kronecker product model by first starting with the basis function represented as $x(u,t)=\sum_{k} \psi_{k}(u,t)\mathrm{x}_{k}$ where each basis function is computed as a product of a spatial and a temporal basis function, $\psi_{i}(\mathrm{u},\mathrm{t})=\psi_{i}^{u}(\mathrm{u})\psi_{j}^{t}(\mathrm{t})$, thus the space-time SPDE[6]. The temporal aspect of the model is based on AR(2) process.

$\frac{\partial}{\partial t}(\mathrm{k}(\mathrm{u})^{2}-\Delta)^{\alpha/2}(\tau(\mathrm{u})\mathrm{x}(\mathrm{u},\mathrm{t}))=\mathrm{w}(\mathrm{u},\mathrm{t}),$ $(\mathrm{u},\mathrm{t})\in\Omega\times R$ (7)

Therefore, the overall non-stationary hierarchical space-time binomial model of the prevalence of stunting was represented as the realization of a spatial-temporal process of stunting among children under the age of five years at the survey location, survey date, significant covariates at sampled locations and date, and the measurement error defined by the Gaussian white noise process. This can simply be denoted as,

$y(u_{i},t)=z(u_{i},t)\beta+\xi(u_{i},t)+\varepsilon(u_{i},t)$ (8)

This equation defines a hierarchical model where $y(\mathrm{u}_{i},t)$ is a realization of a spatial-temporal process that represents risk of stunting at study location $i=1...n$, and year $t.\ldots,T$, $z(\mathrm{u}_{i},t_{j})=(z_{1}(\mathrm{u}_{i},t_{j}),\ldots,z_{p}(\mathrm{u}_{i},t_{j}))$ denotes the vector of $p$ covariates for cluster $u_{i}$ at time $t_{j}$, $\beta=(\beta_{1}\ldots,\beta_{p})'$ is the coefficient vector, $\varepsilon(\mathrm{u}_{i},t_{j})\sim N(0,\sigma_{\varepsilon}^{2})$ is the measurement error defined by the Gaussian white noise process that is uncorrelated both over space and time.

**Validation**

To assess the predictive performance of the final models, we extracted the predicted proportion of children stunting in the surveyed locations and matched with the actual prevalence in surveyed data at the corresponding locations and time. Four performance indices were chosen to evaluate predictive performance and model fit: root-mean-square error (RMSE), mean prediction error (MPE), mean absolute prediction error (MAPE), and the correlation coefficient between the predicted and the observed values. The RMSE is simply the square-root of the mean of the squared difference between the posterior predicted mean and observed value and it is used to measure the accuracy of the model. The MPE provides a measure of the bias of the predictor, the MAPE provides a measure of the mean accuracy of individual predictions, and the correlation coefficient provides a measure of association between the observed data and prediction sets[9]. The correlation between the observed and predicted data was visualized using scatter plots with a least-squares best fitting line and histograms (Figure SI 3).


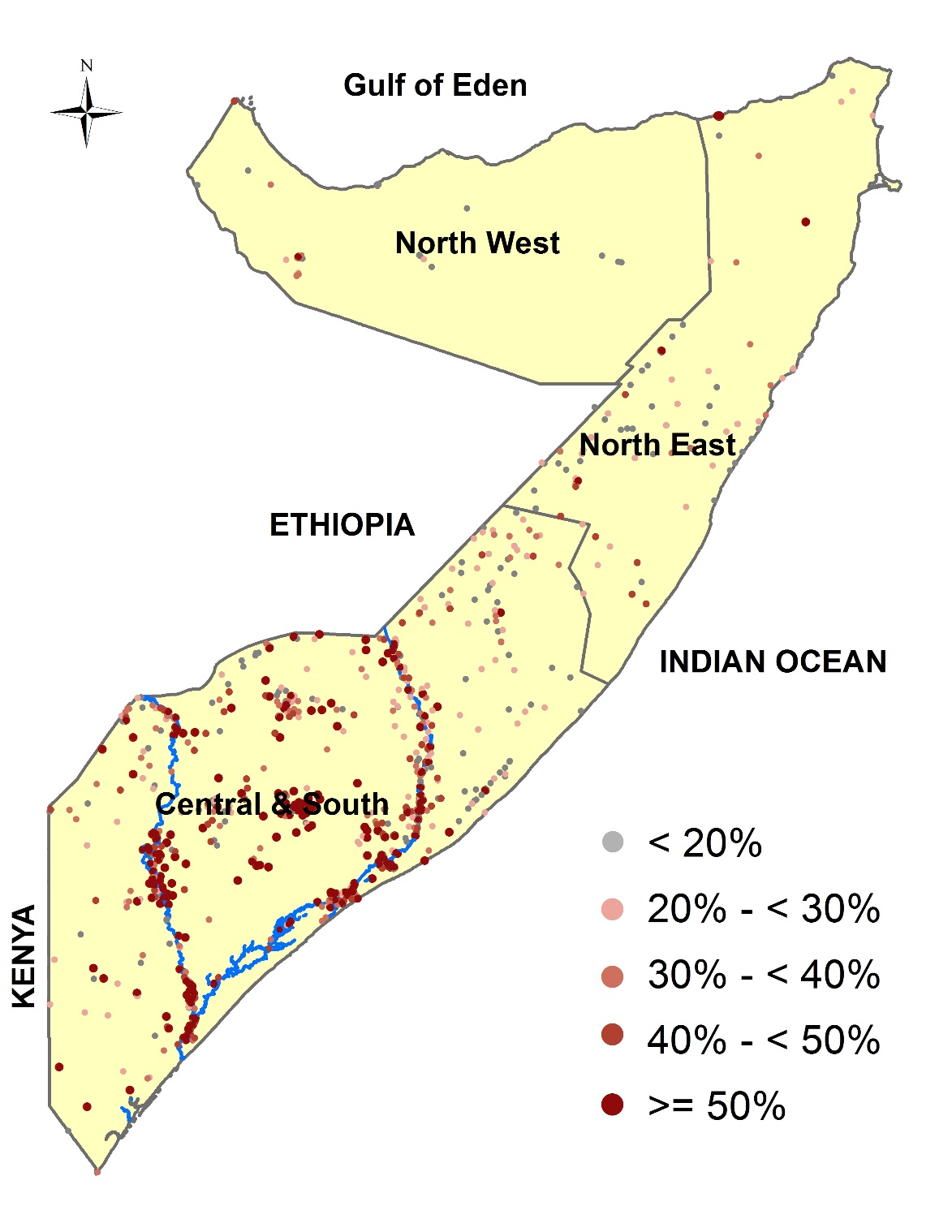


**Figure SI 1a:** Map showing the distribution of clusters sampled for FSNAU nutrition surveys conducted between 2007 and 2010 in Somalia. The country is divided into three main zones: North West, North East and South Central. 78 clusters were sampled in North West zone, 85 clusters in the North East zone and 903 clusters in the South Central. The country’s two main rivers, Juba and Shabelle are located in the South Central zone.

**
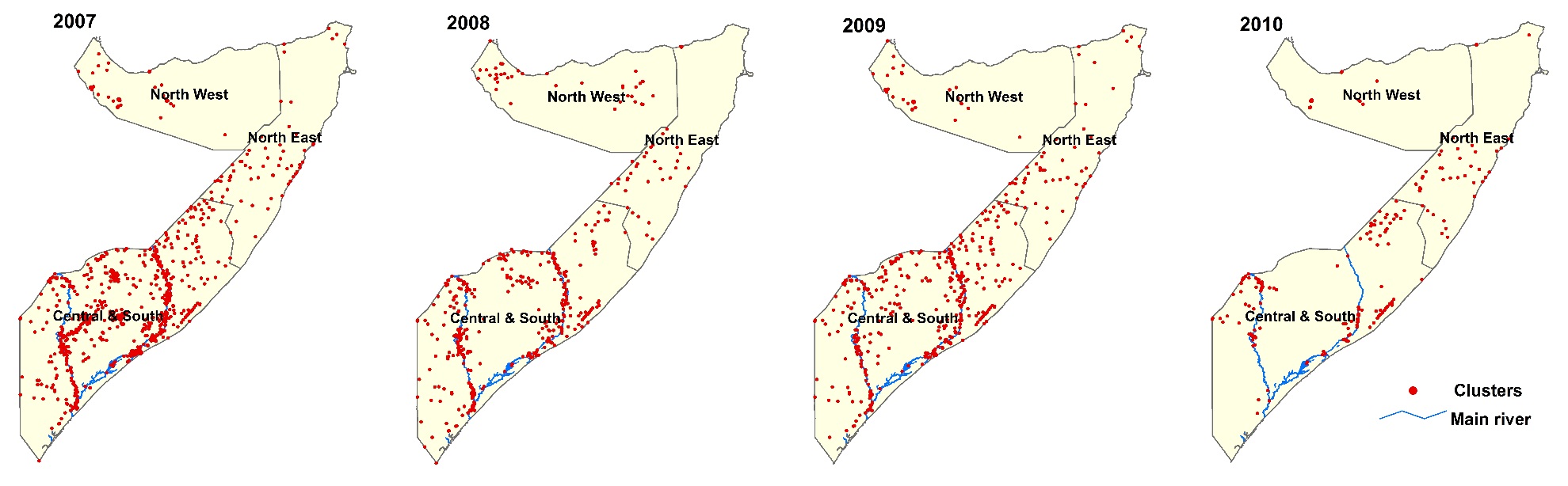
Figure SI 1b:** Patterns of stunting among children under the age of five in Somalia. These data were obtained from Food Security and Nutrition Unit (FSNAU) surveys ranging from the year 2007 to 2010. The data represents a total of 1,066 unique survey locations sampled; 36% of the data was collected in 2007, 27% in 2008, 22% in 2009 and 14% in 2010.

**
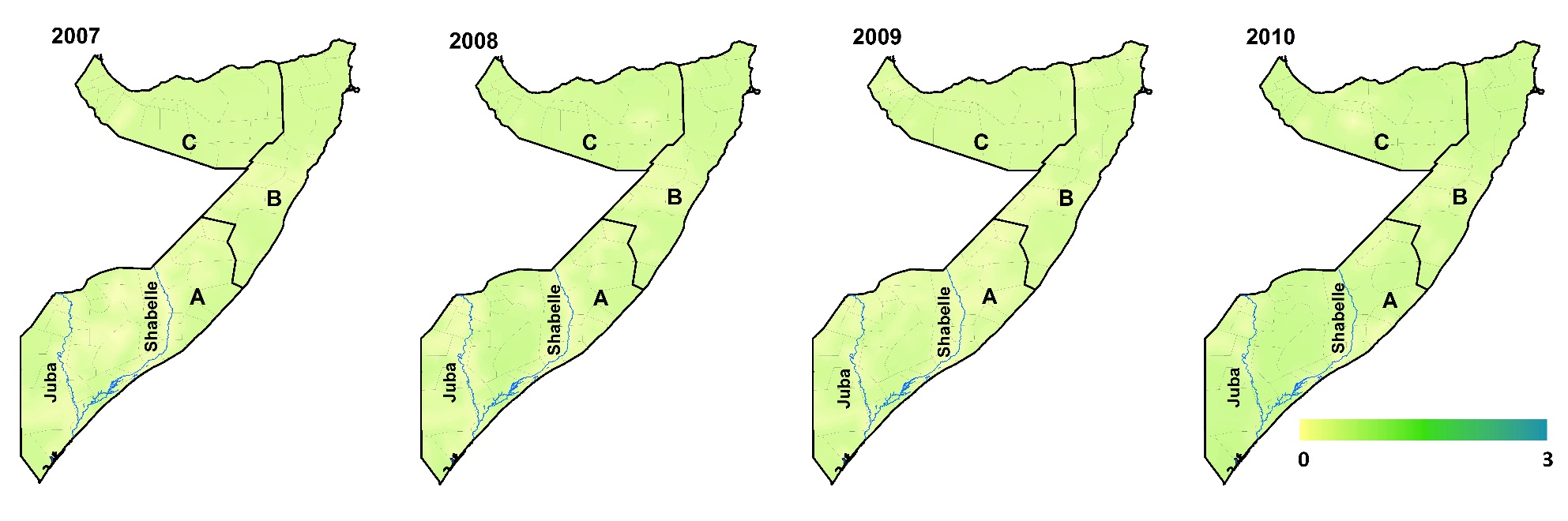
Figure** **SI 2:** Continuous maps at 1 x 1 km spatial resolution of standard deviations from the predicted mean of stunting for the years 2007 to 2010. The maps show that the standard deviations were less than one in all the years of prediction. A=South Central zone, B=North East (Puntland) zone, C=North West (Somaliland) zone.


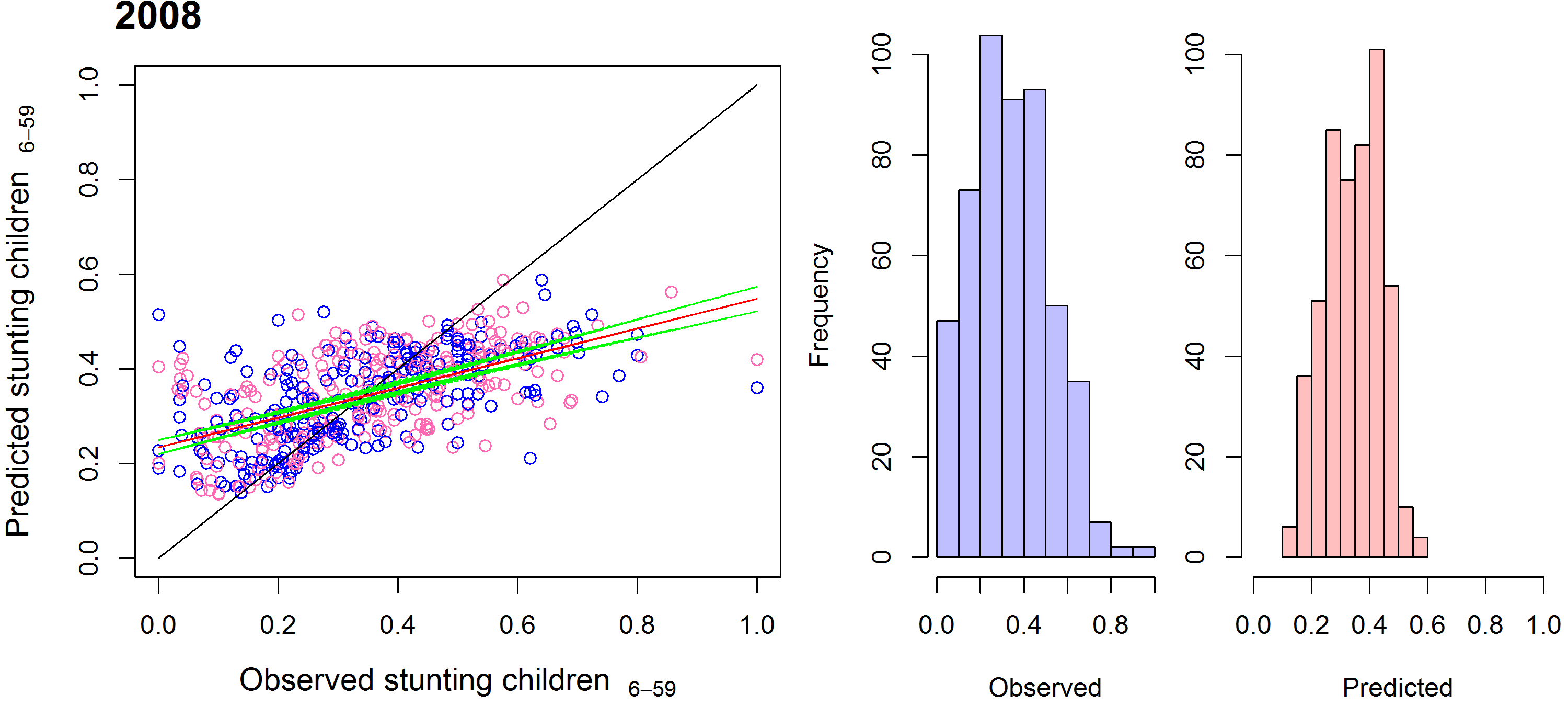


**Figure SI 3a:** Correlation plots of observed and predicted values at cluster level in 2008 for model 1.


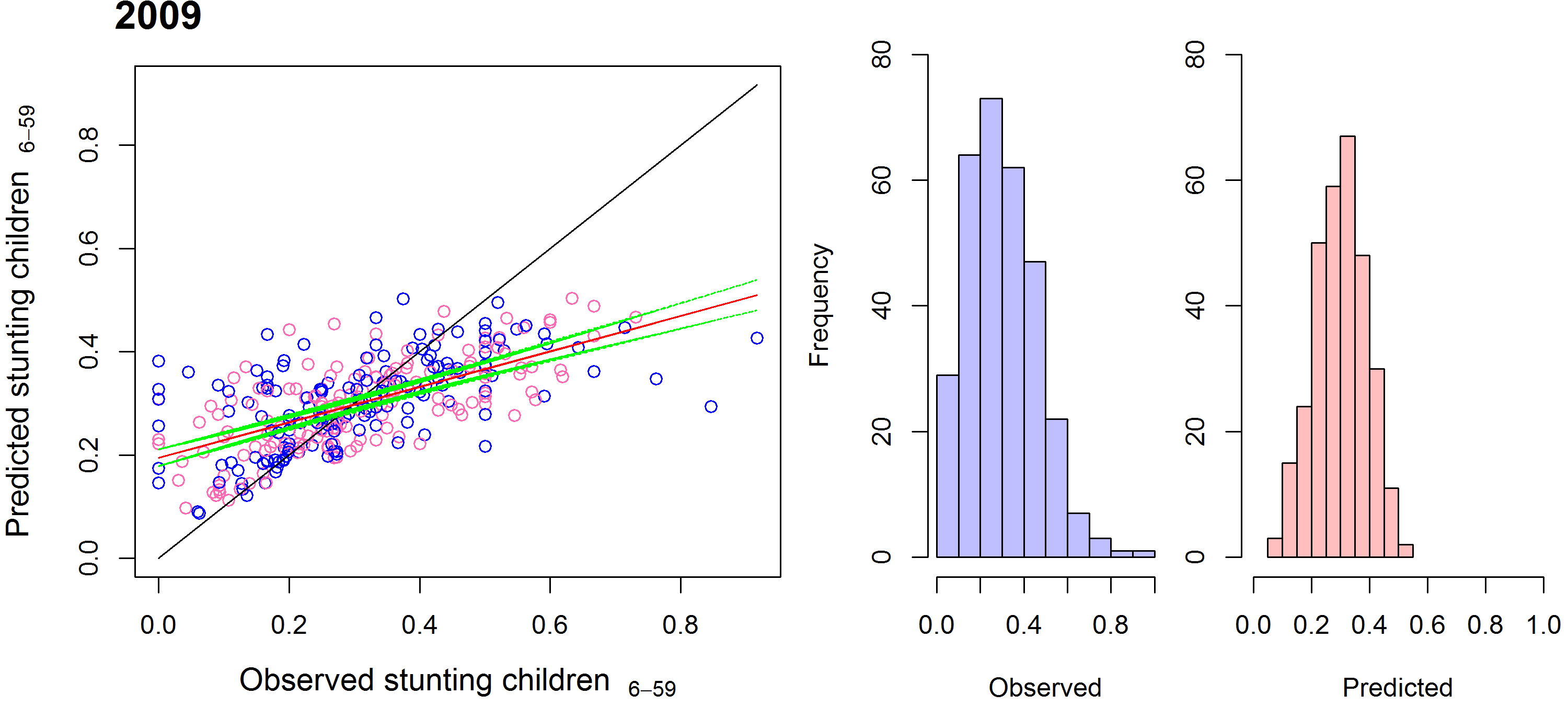


**Figure SI 3b:** Correlation plots of observed and predicted values at cluster level in 2009 for model 1.


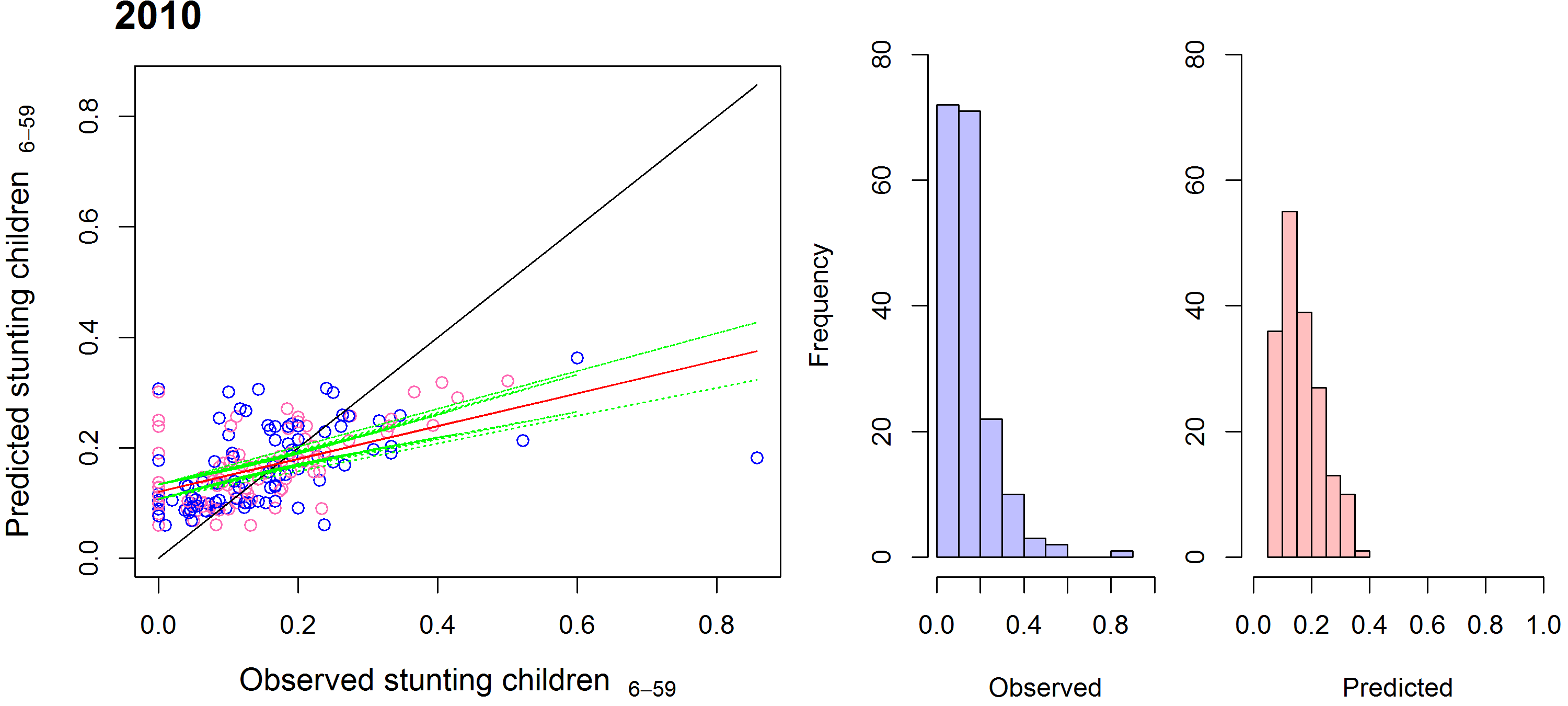


**Figure SI 3c:** Correlation plots of observed and predicted values at cluster level in 2010 for model 1.


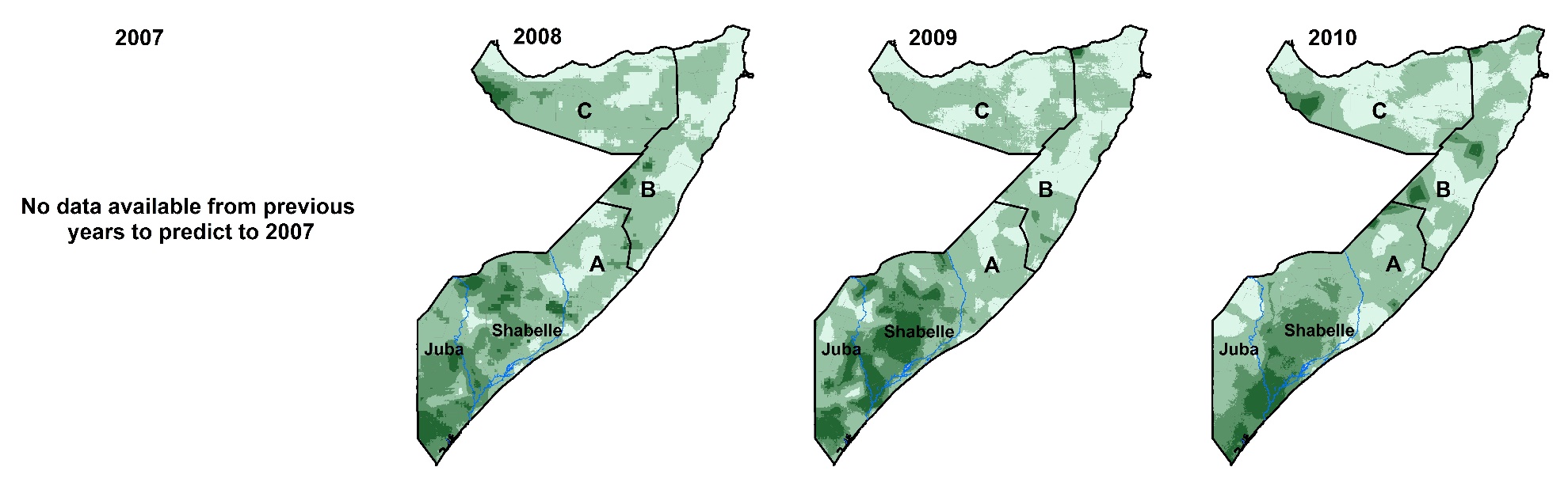


**Figure SI 4a:** Model 1: The predicted 1 x 1 km posterior binned stunting mean maps for 2008 and 2010 for children **aged 6 - 30 months.** Less than 20% represents low prevalence; 20% -< 30%, medium prevalence; 30% - < 40%; high class and >=40% represents the very high prevalence according to the WHO prevalence classification. A=South Central zone, B=North East (Puntland) zone, C=North West (Somaliland) zone. The blue lines represent the two rivers in Somalia (Juba and Shabelle).


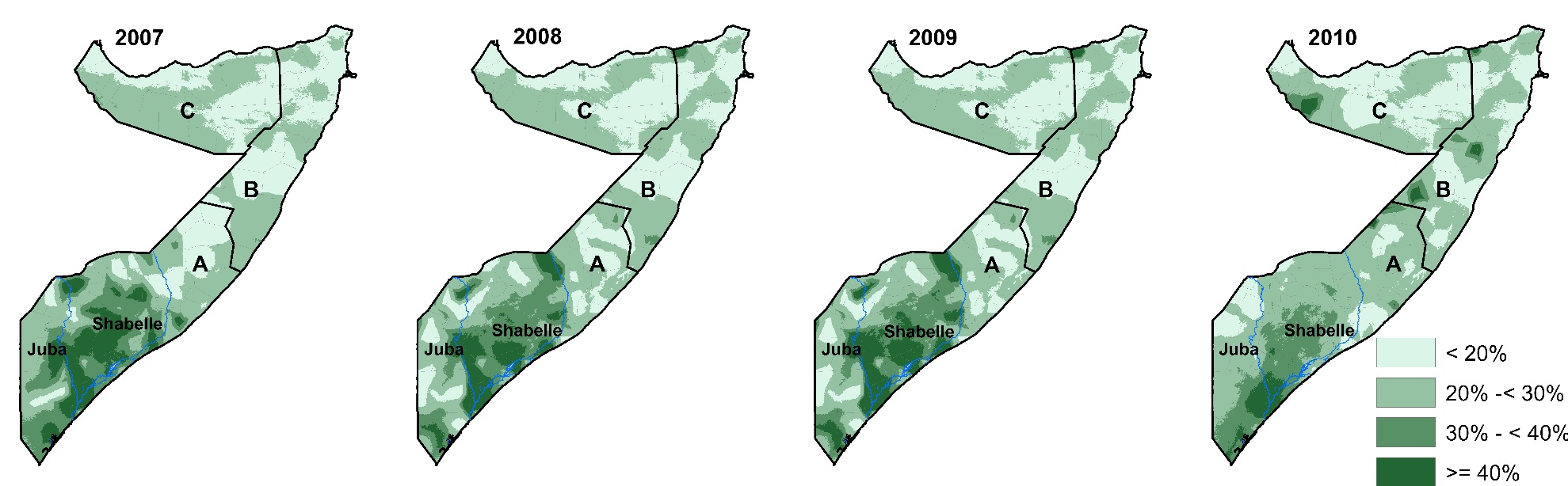


**Figure SI 4b: Model 2:** The predicted 1 x 1 km posterior binned stunting mean maps for 2007 and 2010 for children **aged 6 - 30 months.** Less than 20% represents low prevalence; 20% -< 30%, medium prevalence; 30% - < 40%; high class and >=40% represents the very high prevalence according to the WHO prevalence classification. A=South Central zone, B=North East (Puntland) zone, C=North West (Somaliland) zone. The blue lines represent the two rivers in Somalia (Juba and Shabelle).

# References

1. Murtaugh PA: **Performance of several variable-selection methods applied to real ecological data**. *Ecology Letters* 2009, **12**(10):1061-1068.

2. Scharlemann JorP, Benz D, Hay SI, Purse BV, Tatem AJ, Wint GW, Rogers DJ: **Global data for ecology and epidemiology: a novel algorithm for temporal Fourier processing MODIS data**. *PLoS One* 2008, **3**(1):e1408.

3. Schneider A, Friedl M, Potere D: **A new map of global urban extent from MODIS satellite data**. *Environmental Research Letters* 2009, **4**(4):044003.

4. McLeod A, Xu C: **bestglm: Best Subset GLM**. *URL* [*http://CRAN*](http://CRAN) *R-project org/package= bestglm* 2010.

5. Rue Hav, Martino S, Chopin N: **Approximate Bayesian inference for latent Gaussian models by using integrated nested Laplace approximations**. *Journal of the royal statistical society: Series b (statistical methodology)* 2009, **71**(2):319-392.

6. Lindgren F, Rue H: **Bayesian Spatial and Spatio-temporal Modelling with R-INLA**.

7. Cameletti M, Lindgren F, Simpson D, Rue Hav: **Spatio-temporal modeling of particulate matter concentration through the SPDE approach**. *AStA Advances in Statistical Analysis* 2013:1-23.

8. Ingebrigtsen R, Lindgren F, Steinsland I: **Spatial models with explanatory variables in the dependence structure**. *Spatial Statistics* 2013.

9. Magalhaes RJS, Clements ACA: **Mapping the risk of anaemia in preschool-age children: The contribution of malnutrition, malaria, and helminth infections in West Africa**. *PLoS medicine* 2011, **8**(6).
